# A putative causality of vitamin D in common diseases: A mendelian randomization study

**DOI:** 10.3389/fnut.2022.938356

**Published:** 2022-08-02

**Authors:** Hui Liu, Xudan Shen, Tunan Yu, Yifan Wang, Sheng Cai, Xia Jiang, Xiujun Cai

**Affiliations:** ^1^Zhejiang Provincial Key Laboratory of Laparoscopic Technology, Sir Run Run Shaw Hospital, School of Medicine, Zhejiang University, Hangzhou, China; ^2^Zhejiang Province Key Laboratory of Anti-Cancer Drug Research, Institute of Drug Metabolism and Pharmaceutical Analysis, Zhejiang University, Hangzhou, China; ^3^Department of General Surgery, Sir Run Run Shaw Hospital, School of Medicine, Zhejiang University, Hangzhou, China; ^4^Department of Clinical Neuroscience, Center for Molecular Medicine, Karolinska Institute, Stockholm, Sweden

**Keywords:** vitamin D, Mendelian randomization, Biobank Japan, UK Biobank, FinnGen study

## Abstract

**Backgrounds:**

Vitamin D is considered as a nutrient protecting individuals against an array of diseases based on observational studies. Such a protective effect, however, has not been demonstrated by randomized controlled trials. This study aims to explore a putative causal role of vitamin D in common diseases through a two-sample Mendelian randomization (MR) framework.

**Methods:**

Circulating vitamin D was predicted by 41 genetic variants discovered in European populations. Common diseases were verified through two ways, using information from Japanese patients of Biobank Japan and using information from European patients of FinnGen project. We additionally validated the results by replacing vitamin D-associated instrumental variables (IVs) of European population with that of an independent Japanese population and of an independent Indian population. Inverse-variance weighted method was used as the primary analytical approach while a series of MR methods including MR-Egger regression, weighted median, maximum likelihood, MR-PRESSO and multivariate MR were adopted to guarantee MR model assumptions and to detect horizontal pleiotropy.

**Results:**

Genetically predicted vitamin D was significantly associated with an increased risk of Graves' disease (OR = 1.71, 95%CI: 1.25–2.33, *P* = 0.001) and cataract (OR = 1.14, 95%CI: 1.03–1.28, *P* = 0.016); while with a decreased risk of esophageal cancer (OR = 0.66, 95%CI: 0.46–0.93, *P* = 0.019). This significant causal link between vitamin D and cataract was validated replacing IVs identified in the European population with those from Japanese population. No notable associations of vitamin D with other diseases were observed.

**Conclusions:**

Our findings indicate a potential causal role of vitamin D in common diseases, which needs further validation.

## Introduction

Vitamin D, mainly existing in the body as 25-hydroxyvitamin D [25(OH)D], has been reported to be negatively associated with skeletal diseases such as fractures and falls, as well as various non-skeletal chronic diseases including cancer, cardiovascular diseases, and metabolic disorders (1, 2). However, findings of clinical trials did not support the protective role of vitamin D supplementation in decreasing the risk of falls and fractures (3–5). In addition, a few studies found that higher vitamin D level was related to a higher risk of colon cancer (6) and prostate cancer (7). Gathering above evidences, the role of vitamin D in diverse diseases may be different and complex. Besides, it is uncertain whether there is a putative causal link between vitamin D and diseases, since observational studies may be susceptible to confounding and reverse causation. Therefore, Mendelian randomization (MR), a method using instrumental variable (IV) to unveil the casual relationship, has been comprehensively applied to explain the effect of modifiable exposures (8, 9).

Several MR studies have interrogated the causal role of vitamin D in various diseases. Ong et al. have assessed the association between vitamin D and the susceptibility of 10 cancers and revealed no relationship between vitamin D and cancers, with the exception of ovarian cancer (OR = 0.89, 95%CI:0.82–0.96) (10). Additionally, an earlier phenome-wide MR (MR-PheWAS) analysis integrating the phenome-wide association study (PheWAS) and MR method found there was no evidence of a moderate or large (OR>1.2) causal effect of vitamin D on plenty of health outcomes (11). Yet this MR-PheWAS study used six SNPs as IVs, which only explained 2.84% variance of vitamin D, and did not have enough power to detect small causal effects of vitamin D on diseases. Since an updated genome-wide association study (GWAS) of vitamin D was published, a recent MR study investigated the causal links between genetically predicted vitamin D by 143 variants and 106 diseases/traits, and discovered the protective roles of vitamin D in height, ovarian cancer, multiple sclerosis, and fracture (12). Despite that, all three MR studies identified common diseases in European populations, limiting the generalization to other populations.

As an extension of these evidences, a MR-PheWAS analysis is performed to discover the causal links between vitamin D and common diseases in Biobank Japan cohort. One hundred thirty-eight independent SNPs identified by the most recently published vitamin D GWAS study were used as IVs, which was conducted in a total of 443,734 European individuals (13). In addition, Biobank Japan (BBJ) project is a largest Biobank project with a total of 260,000 patients of non-European population covering 51 common diseases (14). This study aims to provide a comprehensive understanding of the causal role of vitamin D in multiple diseases.

## Materials and methods

### Data for IV-exposure

We retrieved the summary statistics for the effect size of SNPs significantly associated with circulating 25(OH)D concentration from a meta-GWAS of vitamin D involving a GWAS in 401,460 white British subjects from UK Biobank (UKBB) cohort and another independent GWAS in 42,274 European participants (13). Since 25(OH)D level was first log-transformed and then standardized in meta-GWAS analysis, all effect estimates in this MR-PheWAS study corresponded to log-transformed 25(OH)D concentration per standard deviation increase. Totally, 138 conditionally independent SNPs passing genome-wide significance (*P* < 5 × 10^−8^) were identified by GCTA-COJO v.1.91.1 using the meta-GWAS, and used as our exposure of interest (15). The total variance explained by these vitamin D associated SNPs was 4.9%.

In addition, we performed quality control on genetic variants of exposure of interest. The selection criterion was biallelic SNPs with *P* value reaching genome-wide significance (*P* < 5.0 × 10^−8^), minor allele frequency (MAF) >0.01, call rate >95%, linkage disequilibrium r^2^ >0.9, and *P* value for Hardy-Weinberg equilibrium >1.0 × 10^−6^. Besides, the palindromic SNPs that could not verify whether alleles were correctly orientated, and the pleiotropic SNPs which were associated with other traits other than the exposure and outcomes of interest according to GWAS Catalog (accessed on June 2022) were excluded. Detailed information on vitamin D instruments was shown in Supplementary Table 1.

In order to ensure a valid MR result, strong IV is essential element, which was reflected by F-statistics. It is calculated using formula $F=\frac{R^{2}\left(n-1-k\right)}{\left(1-R^{2}\right)k}$, where R^2^ is the proportion of variance explained by the IVs, k refers to the number of IVs, and n indicates the sample size (16). The F-statistics for 25(OH)D level was 203.9, indicating strong IVs (F-statistics > 10) for our exposure of interest.

### Data for IV-outcome

Diseases were identified from BBJ Project, which established a large-scale database containing genomic information (covering 8,712,794 autosomes genetic variants) and diseases status in non-European population (14). BBJ database recruited 260,000 Japanese patients representing 440,000 cases of 51 types of common diseases, where each individual was followed up with an average of 10 years. The variant annotation (SNP rsID, chromosome position, effect and reference allele) and summary-level data (beta coefficients, standard error, and *P* value) for each SNP with 42 diseases were extracted (https://humandbs.biosciencedbc.jp/en/hum0014-v21#42diseases; https://biobankjp.org/), and harmonized the effect estimates and reference alleles between vitamin D GWAS and outcome GWAS. Sample sizes for the 42 diseases ranged from 90,336 to 212,453 (Supplementary Table 2).

Briefly, 42 diseases covered seven categories including thirteen types of cancer (lung cancer, breast cancer, gastric cancer, colorectal cancer, prostate cancer, gallbladder/cholangiocarcinoma, cervical cancer, uterine cancer, esophageal cancer, hematopoietic tumor, liver cancer, ovarian cancer and pancreas cancer), four respiratory tract diseases (bronchial asthma, chronic obstructive pulmonary disease, interstitial lung disease/pulmonary fibrosis and pulmonary tuberculosis), three cardiovascular diseases (arrhythmia, coronary artery disease and heart failure), three chronic liver diseases (chronic hepatitis B, chronic hepatitis C and liver cirrhosis), two cerebrovascular disorders (cerebral aneurysm and cerebral infarction), two eye diseases (cataract and glaucoma), fifteen others disease (atopic dermatitis, drug eruption, endometriosis, epilepsy, Graves' disease, keloid, nephrotic syndrome, osteoporosis, peripheral artery disease, periodontitis, hay fever, rheumatoid arthritis, diabetes mellitus, uterine fibroids and urolithiasis).

### Validation in other populations

Given that the population of exposure was European, whereas the population of outcome was Japanese, we replicated analyses in populations of similar genetic background. With regard to IV-exposure, we extracted summary level data of the associations of five SNPs with serum 25(OH)D concentration from Japanese patients with rheumatoid arthritis (*N* = 1957) (17). Due to the limitation of sample size and the number of valid IVs, we then extracted summary statistics of SNPs associated with 25(OH)D concentration from a GWAS study of vitamin D in Asian Indian diabetic heart study/Sikh diabetes study (AIDHS/SDS) (18). AIDHS/SDS comprised of 3,538 subjects from South Asian population and found 24 putative SNPs significantly associated with 25(OH)D level using a two stage GWAS analysis.

Regarding the IV-outcome, summary statistics of genetic variants associated with the risk of outcome from FinnGen database were used. The FinnGen study is a large personalized medicine project covering 500,000 Finnish biobank participants, and aims to provide the evidence of genomic effect on human health. We extracted relative data from data freeze 7 of the FinnGen study, which consisted of 321,302 individuals with almost 17 million genetic variants detected (https://console.cloud.google.com/storage/browser/finngen-public-data-r7/summary_stats/). In the FinnGen study, summary statistics of five diseases including Graves' disease, senile cataract, other cataract, benign esophageal cancer and malignant esophageal cancer were extracted.

### Statistical analysis

MR analysis, which uses SNPs as IVs, can infer the causal effects of an exposure [25(OH)D concentration] on outcomes (multiple diseases), since SNPs are assumed to be randomly allotted and occurs before the onset of disease. It is crucial to assure valid IVs so that to deduce the right causal link, where three essential assumptions should be satisfied (19): (1) IVs should be associated with the exposure; (2) IVs should not be associated with any confounders of exposure-outcome relationship; (3) only through the exposure of interest can IVs affect the outcome.

To establish the causation of vitamin D with the risk of common diseases, two-sample MR analyses were performed, in which the populations of exposure of interest and outcomes of interest came from two non-overlapping populations. Inverse-variance weighted (IVW) approach was applied as our main MR method, which calculated the ratio of SNP-outcome association to SNP-exposure association and then combined the multiple IVs weighted by the inverse of their variances (20). Cochran's Q test was used to assess the potential heterogeneity and *P* value <0.05 was considered as the existence of heterogeneity. As complements of IVW method, weighted median method and maximum likelihood method were applied to test the robustness of IVW approach. Weighted median method used the inverse of the variance of the ratio estimates as weights and calculated the median as the estimated casual effect, under the condition that at least 50% genetic variants were valid IVs (21). Maximum likelihood method assumed a liner regression between the risk factor and outcome, as well as a bivariate normal distribution for the genetic estimates (22).

The main issue of MR analyses was horizontal pleiotropy which the variant had an effect on outcome outside of its effect on the exposure, herein, two MR methods were adopted. Firstly, MR-Egger regression, a regression approach taking the existence of intercept terms into account, detected the potential pleiotropy by the non-zero intercept under the instrument strength independent of direct effect (InSIDE) assumption (23). Secondly, MR-pleiotropy residual sum and outlier (MR-PRESSO) method, which identified a specific SNP by the difference between calculated and expected values of the residual sum of squares using a leave-one-out approach, detecting horizontal pleiotropy by a global test (P <1.0 × 10^−6^) and correcting the estimates excluding SNP outliers (24).

Moreover, we conducted multivariable MR (MVMR) analysis, which provided estimations of independent direct effects of multiple and potentially related exposures [i.e., 25(OH)D level and body mass index (BMI)] on an outcome (25). Considering that 25(OH)D levels are closely related with BMI, summary statistics for BMI estimated from UKBB participants of GIANT Consortium were used in the MVMR model (26).

Statistical significance was regarded as *P* value <0.05 according to IVW method, while directional consistency and significance were verified by other MR methods. Bonferroni correction method was used to account for multiple comparisons, where we set a more conservative *P* value threshold using 0.05 divided by the number of outcomes (0.05/42 ≈ 1.0 × 10^−3^) to avoid false positive results taken by an insufficiently rigorous threshold.

## Results

**Figure 1:**
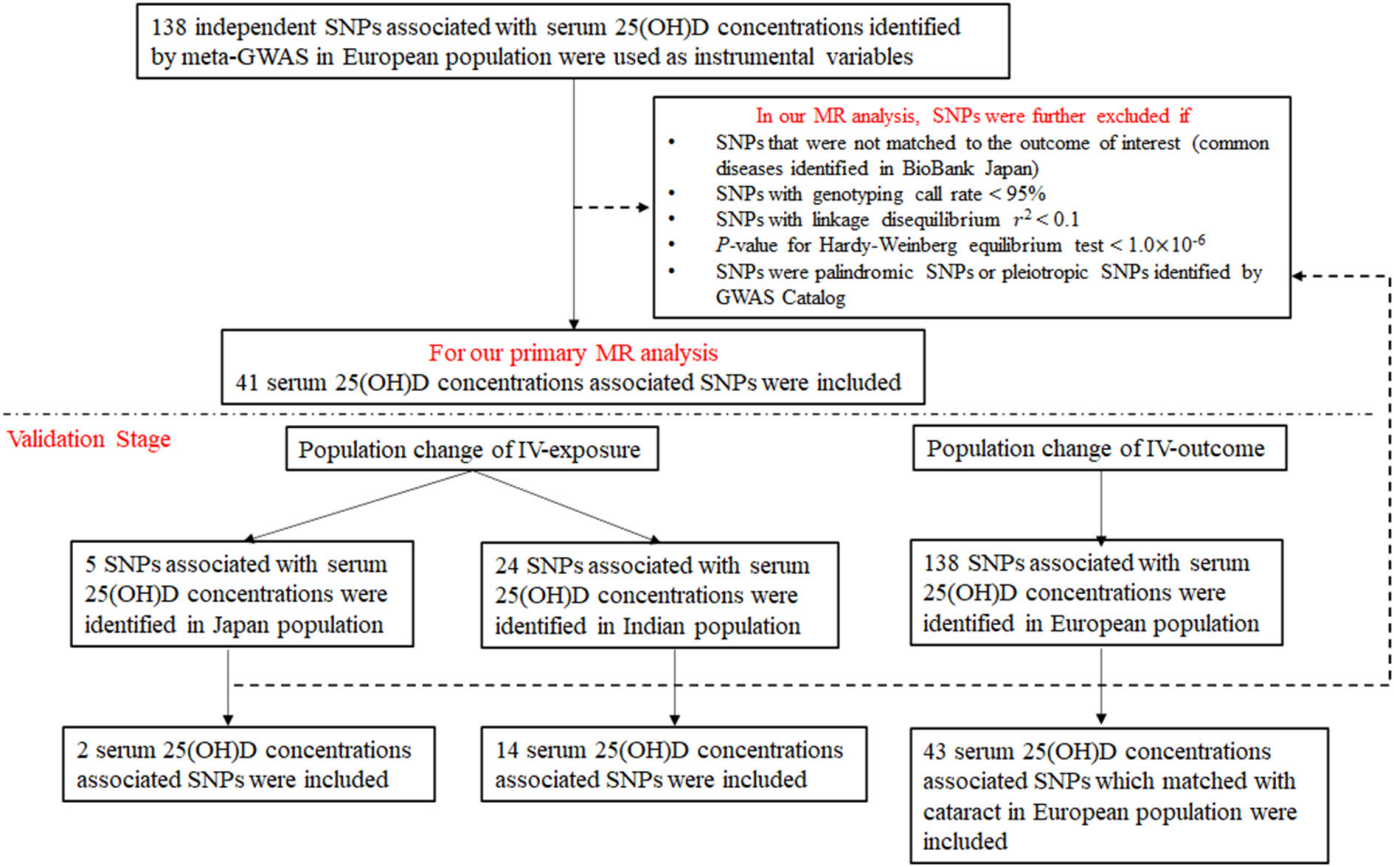
Flowchart on the selection of instrumental variables.

The procedure of SNPs selection is displayed in Figure 1. Among the 138 genetic variants significantly associated with circulating 25(OH)D concentrations, 41 SNPs that were successfully matched to common diseases in BBJ project were included in our primary MR analysis. According to IVW approach, we found vitamin D was associated with a higher risk of Graves' disease (OR = 1.71, 95%CI: 1.25–2.33) and cataract (OR = 1.14, 95%CI: 1.03–1.28); while with a lower risk of esophageal cancer (OR = 0.66, 95%CI: 0.46–0.93). After multiple comparisons correction, the causal relationship between vitamin D and Graves' disease remained significant (*P* = 0.001). All results remained directional consistent and significant in other MR methods including weighted median method, maximum likelihood method, MR-Egger regression and MR-PRESSO (Table 1). Moreover, we found no obvious heterogeneity according to IVW method (*P* > 0.05), neither obvious pleiotropy based on MR-Egger regression (*P* > 0.05) and MR-PRESSO method (*P* > 1.0 × 10^−6^).

**Table 1:** Causal link between genetically predicted vitamin D in European population and diseases identified in individuals from Biobank Japan.

**Disease**	**Method**	**N of IVs**	**OR (95%CI)**	***P***	P^*^
Graves' disease	Inverse-variance weighted	41	1.71 (1.25–2.33)	0.001	0.657
	Weighted median	41	1.83 (1.18–2.83)	0.007	
	Maximum likelihood	41	1.71 (1.24–2.38)	0.001	
	MR-Egger	41	2.10 (1.29–3.42)	0.005	0.264
	MR-PRESSO	41	1.71 (1.25–2.33)	0.002	0.696
Cataract	Inverse-variance weighted	41	1.14 (1.03–1.28)	0.016	0.425
	Weighted median	41	1.14 (0.99–1.32)	0.076	
	Maximum likelihood	41	1.14 (1.03–1.27)	0.014	
	MR-Egger	41	1.16 (0.98–1.36)	0.089	0.851
	MR-PRESSO	41	1.14 (1.03–1.28)	0.020	0.454
Esophageal cancer	Inverse-variance weighted	41	0.66 (0.46–0.93)	0.019	0.921
	Weighted median	41	0.66 (0.38–1.12)	0.123	
	Maximum likelihood	41	0.66 (0.43–1.00)	0.050	
	MR-Egger	41	0.61 (0.33–1.13)	0.124	0.733
	MR-PRESSO	41	0.66 (0.46–0.93)	0.024	0.943

* *P indicates P value of heterogeneous from IVW approach, or P value of intercept from MR-Egger regression, or P value from Mendelian randomization pleiotropy residual sum and outlier (MR-PRESSO) global test*.

Findings of the casual links between vitamin D and multiple diseases were shown in Supplementary Table 3. Despite significant association between vitamin D and atopic dermatitis was noted using MR-Egger regression (OR = 0.40, 95%CI: 0.24–0.66) and weighted median method (OR = 0.56, 95%CI: 0.35–0.89), non-significant relationship was observed using IVW. According to our criteria of statistical significance, we regarded the above result as null association. According to the results of MR-PRESSO method (Supplementary Table 4), two outcomes including coronary artery disease and diabetes mellitus indicated the presence of pleiotropy (*P* < 1.0 × 10^−6^), however, the results did not change after removing outlier SNPs. Since the number of SNPs used as IVs dramatically decreased in the MVMR model, the causal associations of vitamin D with the risk of Graves' disease, cataract and esophageal cancer were attenuated toward null (Supplementary Table 5).

Considering the population heterogeneity between exposure and outcomes of interest, we firstly sought summary statistics of genetic variants predicting 25(OH)D concentration in Japanese population. As presented in Table 2, the strength of the causal estimate between vitamin D and cataract was stronger, the odds ratio calculated using the IVW method was 1.94 (95%CI: 1.90–1.98). Yet, the causal associations of vitamin D with the risk of Graves' disease and esophageal cancer were attenuated toward null. Since there were only 2 SNPs regarded as IVs in the Japanese population, the results were further replicated in another Asian population (Indian) that provided more valid IVs to genetically predict vitamin D. Unfortunately, we did not observe any statistically significant causal association of vitamin D with the risk of either Graves' disease or cataract in the Indian population. Nevertheless, an increased risk of esophageal cancer was found in this population (OR = 1.67, 95%CI:1.08–2.57). Results of the causal estimates between vitamin D and common diseases identified in Asian populations were shown in Supplementary Tables 6, 7.

**Table 2:** Causal link between genetically predicted vitamin D in Asian population and diseases identified in individuals from Biobank Japan.

**Disease**	**Data source of vitamin D**	**N of IVs**	**OR (95%CI)**	***P***	P^*^
Graves' disease	Japanese population	2	1.34 (0.040–50.66)	0.875	0.040
	Indian population	14	1.05 (0.74–1.49)	0.773	0.374
Cataract	Japanese population	2	1.94 (1.90–1.98)	<0.001	0.973
	Indian population	14	1.08 (0.98–1.19)	0.106	0.735
Esophageal cancer	Japanese population	2	1.00 (0.08–13.13)	0.998	0.253
	Indian population	14	1.67 (1.08–2.57)	0.020	0.438

* *P indicates P value of heterogeneous from IVW approach*.

Regarding the outcome of interest, summary statistics of Japanese population from Biobank Japan were replaced with European population from FinnGen study for additional validation analysis. As noted in Table 3, the causal links between vitamin D and different outcomes identified in European population were in general null.

**Table 3:** Causal link between genetically predicted vitamin D in European population and diseases identified in European individuals from FinnGen study.

**Disease**	**Method**	**N of IVs**	**OR (95%CI)**	***P***	P^*^
Graves' disease	Inverse-variance weighted	43	1.34 (0.91–1.98)	0.136	0.132
	Weighted median	43	1.25 (0.77–2.05)	0.370	
	Maximum likelihood	43	1.34 (0.95–1.90)	0.096	
	MR-Egger	43	1.35 (0.82–2.20)	0.241	0.980
Senile cataract	Inverse-variance weighted	43	0.99 (0.90–1.10)	0.911	0.030
	Weighted median	43	0.94 (0.83–1.08)	0.389	
	Maximum likelihood	43	0.99 (0.91–1.08)	0.893	
	MR-Egger	43	1.01 (0.89–1.15)	0.823	0.601
Other cataract	Inverse-variance weighted	43	0.84 (0.72–0.97)	0.021	0.060
	Weighted median	43	0.73 (0.59–0.89)	0.002	
	Maximum likelihood	43	0.84 (0.73–0.95)	0.007	
	MR-Egger	43	0.79 (0.65–0.95)	0.019	0.322
Benign esophageal cancer	Inverse-variance weighted	43	0.90 (0.42–1.95)	0.793	0.933
	Weighted median	43	1.52 (0.44–5.25)	0.507	
	Maximum likelihood	43	0.90 (0.36–2.27)	0.827	
	MR-Egger	43	1.43 (0.45–4.52)	0.546	0.197
Malignant esophageal cancer	Inverse-variance weighted	43	1.00 (0.48–2.08)	0.998	0.185
	Weighted median	43	0.77 (0.32–1.86)	0.563	
	Maximum likelihood	43	1.00 (0.51–1.96)	0.997	
	MR-Egger	43	0.68 (0.27–1.67)	0.401	0.163

* *P indicates P value of heterogeneous from IVW approach, or P value of intercept from MR-Egger regression*.

## Discussion

This phenome-wide MR study explored the causal role of vitamin D in a large quantity of common diseases, including cancers, cardiovascular disease, respiratory tract disease, eye disease, and chronic liver disease, which were identified among more than 200,000 Japanese participants. We found the potential causal associations of vitamin D with the risk of Graves' disease, cataract and esophageal cancer in individuals from Biobank Japan. Moreover, the causal link between vitamin D and Graves' disease remained significant at a more conservative *P* value threshold (*P* = 0.001). In addition, the significant causal relationship of vitamin D with the risk of cataract was verified by replacing vitamin D instruments of European population with Japanese population.

Our findings refuted the protective role of vitamin D suggested by previous observational studies. Vitamin D deficiency has been considered to play a role in the occurrence and development of Graves' disease, which was indicative of lower circulating 25(OH)D level for Graves' disease patients (55.0 ± 23.2 vs. 87.2 ± 27.6 nmol/L) (27, 28). Another case-control study including 51 cases with Graves' disease and 51 healthy controls found there was no significant difference of 25(OH)D levels (81.77 ± 5.60 vs. 83.49 ± 6.24 nmol/L) (29). Although these results contradicted with ours which indicated vitamin D was associated with an 71% increased risk of Graves' disease (OR = 1.71, 95%CI: 1.25–2.33), further large-scale studies need to be performed.

Previous investigations considered vitamin D had a protective role in preventing diseases, such as cataract and esophageal cancer. A case-control study discovered mean 25(OH)D level in cataract patients were significantly lower than that in control group (7.6 ± 5.5 vs. 18.5 ± 9.6 ng/mL) (30). Another study based on Korean National Health and Nutrition Examination Survey found the inverse relationship between serum 25(OH)D level and the risk of nuclear cataract where the odd ratio was 0.86(95%CI:0.75–0.99) (31). On the contrary, there was no notable association of vitamin D and nuclear cataract among women of all ages in the Carotenoids in Age-Related Eye Disease Study (OR = 0.97, 95%CI: 0.65–1.45) (32). As reported by the IVW method, we found vitamin D to be associated with an increased 14% risk of cataract (OR = 1.14, 95%CI: 1.03–1.28), which was in accordance with a previous MR study (12). It indicated an increased risk of cataract using 60 SNPs genetically predicting vitamin D in a population from UK Biobank, where the odds ratio was 1.01(95%CI: 1.00–1.02) with nominal statistical significance.

Previous investigations demonstrated that vitamin D had no association with the risk of esophageal cancer. A MR study using six SNPs associated with vitamin D level as IVs found the odds ratio for esophageal cancer was 0.68 (95%CI:0.39–1.19) among 4,112 patients with esophageal cancer and 17,159 controls (33). Using a larger set of variants associated with vitamin D level (76 SNPs) identified by UKBB, Ong et al. did not find vitamin D was to be a causal risk factor for esophageal cancer (OR = 0.97, 95%CI: 0.78–1.20) (10). Yet our study indicated that genetically predicted vitamin D was associated with a 34% decreased risk of esophageal cancer.

In general, the null findings for multiple health outcomes including most types of cancers, cardiovascular and cerebrovascular disease, autoimmune disease, and metabolic disease of our MR analysis were in accordance with previous MR results. On the contrary, there are some disparities. For example, a protective effect of vitamin D predicted by 143 independent loci on ovarian cancer in participants from UKBB was found by Ye et al. with the odds ratio of 0.96 (95%CI: 0.94–0.98) (12), which was not confirmed in our study (OR = 0.77, 95%CI:0.54–1.10). The discrepancies may be explained by different IVs used to genetically predict vitamin D level, population heterogeneity from both exposure and outcome of interest, or different threshold of statistical significance.

According to traditional theory, the protective role of vitamin D was mainly played by regulation of the immune system, such as activation of cytolytic T cells, and up-regulation of the anti-microbial peptide CAMP or the plasma membrane-anchored glycoprotein CD14 (34). Nevertheless, one possible reason for the raised risk of cataract with high vitamin D level was that a higher vitamin D concentration yields to a stronger absorption of dietary calcium, a risk factor for cataract (35).

A major strength of our investigation was performing a phenome-wide MR study with a large number of genetic variants and common diseases in a large-scale Japanese population. Yet several limitations cannot be ruled out. Firstly, we performed a large amount of health outcomes in this MR study, which increased the risk of false positive findings. Thus, we defined the threshold of statistical significance as 1.0 × 10^−6^ using Bonferroni correction method. Secondly, 41 independent loci associated with circulating 25(OH)D concentration was used as IVs, which might violate the exclusion restriction assumption of a valid MR analysis, because some IVs may have an effect on diseases not mediated by vitamin D. In order to test the horizontal pleiotropy, two MR methods including MR-Egger regression and MR-PRESSO method were conducted, where the results by these methods were consistent with that by the IVW approach. In addition, we assumed that the relationships between vitamin D and common diseases were linear, however, the shape of association between vitamin D and disease such as prostate cancer (36), cardiovascular disease and all-cause mortality (37) was non-linear. Last but not least, two sample populations of exposure and outcomes of interest came from different ancestry, where exposure was from white British but outcome was from Japanese. Although this met the non-overlap sample requirement of two sample MR method, it may introduce the population heterogeneity and limit the generalizability of results. However, the significant causal link between vitamin D and cataract remained by replicating the analyses in a population which exposure and outcome were both from Japanese. Herein, a large-scale phenome-wide MR study composed with mixed populations was warranted.

In conclusion, this MR study indicated a potential causal relationship between vitamin D and multiple diseases, and a more obvious causal link between vitamin D and Graves' disease. It may have implications for disease prevention; however, this causal link needs to validate or refute in a large prospective study.

## Data availability statement

The original contributions presented in the study are included in the article/Supplementary material, further inquiries can be directed to the corresponding authors.

## Ethics statement

Ethical review and approval was not required for the study on human participants in accordance with the local legislation and institutional requirements. Written informed consent for participation was not required for this study in accordance with the national legislation and the institutional requirements.

## Author contributions

HL, XJ, and XC designed the study. HL, TY, and YW did literature research and collected data. HL, XS, and SC undertook analyses and interpreted the results. HL and XS wrote the first draft of the manuscript. XJ and XC had primary responsibility for final content. All authors reviewed the manuscript. All authors contributed to the article and approved the submitted version.

## Funding

This study was supported by National Natural Science Foundation of China (No. 82103807).

## Conflict of interest

The authors declare that the research was conducted in the absence of any commercial or financial relationships that could be construed as a potential conflict of interest.

## Publisher's note

All claims expressed in this article are solely those of the authors and do not necessarily represent those of their affiliated organizations, or those of the publisher, the editors and the reviewers. Any product that may be evaluated in this article, or claim that may be made by its manufacturer, is not guaranteed or endorsed by the publisher.
